# Neonates Born Through Meconium-stained Amniotic Fluid among Deliveries in a Tertiary Care Centre: A Descriptive Cross-sectional Study

**DOI:** 10.31729/jnma.7917

**Published:** 2022-12-31

**Authors:** Sabina Shrestha, Manoj Pokhrel, Sunil Raja Manandhar

**Affiliations:** 1 Department of Pediatrics, Kathmandu Medical College and Teaching Hospital, Sinamangal, Kathmandu, Nepal; 2 Department of Obstetrics and Gynaecology, Kathmandu Medical College and Teaching Hospital, Sinamangal, Kathmandu, Nepal

**Keywords:** *meconium*, *neonate*, *Nepal*

## Abstract

**Introduction:**

The mortality and morbidity of meconium aspiration syndrome in neonates born with meconium-stained amniotic fluid in developing countries are still high. In Nepal, few studies have estimated the prevalence of meconium-stained amniotic fluid among newborns. The study aimed to find out the prevalence of neonates born through meconium-stained amniotic fluid among deliveries in a tertiary care centre.

**Methods:**

This descriptive cross-sectional study was conducted among neonates born in a tertiary care centre from November 2021 to March 2022. Neonates born during the study period with meconium-stained amniotic fluid were studied. Ethical approval was obtained from the Institutional Review Committee. Convenience sampling method was used. With permission from the Department of the Neonatal Intensive Care Unit, the data were collected and entered in a Microsoft Excel sheet. Point estimate and 95% Confidence Interval were calculated.

**Results:**

Among 576 neonates, the prevalence of neonates born through meconium-stained amniotic fluid was 77 (13.37%) (10.59-16.15, 95% Confidence Interval).

**Conclusions:**

The prevalence of neonates born through meconium-stained amniotic fluid was found to be similar to other studies done in similar settings.

## INTRODUCTION

Respiratory distress is one of the frequent emergencies and the most common causes of mortality and morbidity in newborns. The common causes of respiratory distress are Transient Tachypnea of newborns, Hyaline Membrane Disease and Meconium Aspiration Syndrome.^1^

The prevalence of respiratory distress in a tertiary care centre in Nepal was found to be 6.55% among the total live births and 30.83% among the admitted cases of the Neonatal Intensive Care Unit (NICU) with an estimated mortality of 4.50%.^2^

If the foetal meconium passes into the amniotic fluid then it is called Meconium Stained Amniotic Fluid (MSAF) and usually occurs in approximately 15-20% of term pregnancies and 30-40% of the post-term pregnancies.^3,4^

The study aimed to find out the prevalence of neonates born through meconium-stained amniotic fluid among deliveries in a tertiary care centre.

## METHODS

A descriptive cross-sectional study was conducted among neonates born in Kathmandu Medical College Teaching Hospital (KMCTH) from November 2021 to March 2022. Ethical approval was obtained from the Institutional Review Committee of KMCTH. Neonates born during the study period with meconium-stained amniotic fluid were studied. The inclusion criteria were all newborns born in KMCTH within the study period. The exclusion criteria were patients whose parents did not give consent for the study, neonates with birth injuries, neonates with congenital malformations, multiple gestations and preterm babies. Convenience sampling method was used. The sample size was calculated using the given formula:


$$\begin{array}{ll} \text{n} & ={\text{Z}}^{2}\times \frac{\text{p}\times \text{q}}{{\text{e}}^{\text{2}}}\\ & =1.96^{2}\times \frac{0.50\times 0.50}{0.05^{2}}\\ & =385 \end{array}$$

Where,

n = minimum required sample size,
Z = 1.96 at 95% Confidence Interval (CI),
p = prevalence taken as 50% for maximum sample size calculation
q = 1-p
e = margin of error, 5%

The minimum sample size calculated was 385. However, the final sample size taken was 576.

Data were entered in Microsoft Excel sheet. Point estimate and 95% CI were calculated.

## RESULTS

Among 576 neonates, the prevalence of neonates born through meconium-stained amniotic fluid was 77 (13.37%) (10.59-16.15, 95% CI). Out of 77 meconium-stained liquor, 27 (35.06%) had thin meconium (Table 1).

**Table 1:** Types of meconium stained liquor (n= 77).

Meconium-stained liquor	n (%)
Thin	27 (35.06)
Moderate	24 (31.17)
Thick	26 (33.77)

Thirty-eight (49.35%) women were nulliparous among 77 women who gave birth to neonates who had meconium-stained amniotic fluid. Low-segment Caesarean Section was the mode of delivery in 50 (64.93%) women (Table 2).

**Table 2:** Parity status, mode of delivery, age group of women giving birth to babies with meconium-stained liquor (n= 77).

Parity status	n (%)
Nullipara	38 (49.35)
Multipara	9 (50.65)
**Mode of delivery**	
Normal vaginal delivery	26 (33.77)
Vacuum delivery	1 (1.30)
Low-segment Caesarean Section	50 (64.93)
**Age group of pregnant women (years)**	
19-24	15 (19.49)
25-29	38 (49.35)
30-34	19 (24.67)
35-39	5 (6.49)

Among 77 neonates born through meconium-stained amniotic fluid, 8 (10.39%) developed meconium aspiration syndrome (Table 3).

**Table 3:** Diagnoses neonates with meconium-stained amniotic fluid (n= 77).

Diagnoses	n (%)
Respiratory distress	5 (6.49)
Meconium aspiration syndrome	8 (10.39)
Pneumothorax	1 (1.30)
Convulsion	1 (1.30)
Sepsis	2 (2.60)
Hypoxic-ischaemic encephalopathy	1 (1.30)
Hypoglycemia	1 (1.30)
Hyperbilirubinemia	3 (3.90)

Premature rupture of membranes was seen in 6 (7.79%) mothers of neonates born through a meconium-stained amniotic fluid (Table 4).

**Table 4:** Maternal complications among mothers of neonates born through a meconium-stained amniotic fluid (n= 77).

Complications	n (%)
Premature rupture of membrane	6 (7.79)
Oligohydramnios	1 (1.30)
Pregnancy-induced hypertension	3 (3.90)
Gestational diabetes mellitus	5 (6.49)
Obstetric cholestasis	1 (1.30)

## DISCUSSION

In our study, the prevalence of meconium-stained amniotic fluid among neonates was found in 77 (13.37%) deliveries. The incidence of MAS was high in Nepal with an overall incidence of about 2.0 per 1000 live births.^11^ Studies conducted in Nepal have revealed the prevalence of MSAF about 6.5%-14.6%,^8,9,12^ among all live deliveries, the prevalence of MAS among MSAF about 6.6% to 8.6%,^8,12^ and neonatal mortality from MAS about 5.4% to 11.3%.^8,9,12^ The study conducted in India found the prevalence of MAS among MSAF as 7.7% to 21.5%,^4-6^ and mortality among MAS as high as 13.8%.^4-6^ The most common complication of MAS is Respiratory distress and other morbidities were convulsions, sepsis, shock, hypoxic-ischemic encephalopathy, hyperbilirubinemia and hypoglycemia.^13^

Several studies conducted in Nepal have revealed the prevalence of MSAF about 6.5%-14.6%,^8,9,12^ among all live deliveries, the prevalence of MAS among MSAF about 6.6% to 8.6%,^8,12^ and neonatal mortality from MAS about 5.4% to 11.3%.^8,9,12^

MSAF is an important determinant for both maternal and neonatal morbidity and mortality. The complicated MSAF needs prolonged admission of neonates in the NICU.^4-6,8-12^ The cost of care and utilisation of critical care services are frequent targets of concern for the healthcare system in a resource-limited setting.

Meconium-stained amniotic fluid not only poses risk to the newborn but also significantly increases the rate of maternal complications such as meconium-laden amniotic fluid embolism, intrapartum chorioamnionitis, puerperal endometritis, wound infection, increased risk of operative delivery and its complication.^13,14^ The perinatal morbidity and mortality related to MSAF can be decreased if major risk factors are recognized early and closely monitoring of the labour and careful decisions are made about the timing and mode of delivery.^14^

With the improvement of obstetric practices and perinatal care, the incidence of MAS and other causes of Respiratory distress is decreasing in developed countries.^3,7^ However, Meconium aspiration syndrome (MAS) is still an important cause of neonatal mortality and morbidity in neonatal intensive care units (NICUs) in developing countries.^4-6,8-12^

This study is a single institution-based descriptive cross-sectional study, the results might not be completely generalizable in other settings. So, a study design with a higher level of evidence is recommended for future studies. There may be non-response data as data will be collected from the patient party. There may also be information bias and respondent bias.

## CONCLUSIONS

The prevalence of neonates born through meconium-stained amniotic fluid among deliveries was similar to other studies done in similar settings.
